# Enabling Early Health Care Intervention by Detecting Depression in Users of Web-Based Forums using Language Models: Longitudinal Analysis and Evaluation

**DOI:** 10.2196/41205

**Published:** 2023-03-24

**Authors:** David Owen, Dimosthenis Antypas, Athanasios Hassoulas, Antonio F Pardiñas, Luis Espinosa-Anke, Jose Camacho Collados

**Affiliations:** 1 School of Computer Science and Informatics Cardiff University Cardiff United Kingdom; 2 Centre for Medical Education School of Medicine Cardiff University Cardiff United Kingdom; 3 Centre for Neuropsychiatric Genetics and Genomics School of Medicine Cardiff University Cardiff United Kingdom

**Keywords:** mental health, depression, internet, natural language processing, transformers, language models, sentiment

## Abstract

**Background:**

Major depressive disorder is a common mental disorder affecting 5% of adults worldwide. Early contact with health care services is critical for achieving accurate diagnosis and improving patient outcomes. Key symptoms of major depressive disorder (depression hereafter) such as cognitive distortions are observed in verbal communication, which can also manifest in the structure of written language. Thus, the automatic analysis of text outputs may provide opportunities for early intervention in settings where written communication is rich and regular, such as social media and web-based forums.

**Objective:**

The objective of this study was 2-fold. We sought to gauge the effectiveness of different machine learning approaches to identify users of the mass web-based forum Reddit, who eventually disclose a diagnosis of depression. We then aimed to determine whether the time between a forum post and a depression diagnosis date was a relevant factor in performing this detection.

**Methods:**

A total of 2 Reddit data sets containing posts belonging to users with and without a history of depression diagnosis were obtained. The intersection of these data sets provided users with an estimated date of depression diagnosis. This derived data set was used as an input for several machine learning classifiers, including transformer-based language models (LMs).

**Results:**

Bidirectional Encoder Representations from Transformers (BERT) and MentalBERT transformer-based LMs proved the most effective in distinguishing forum users with a known depression diagnosis from those without. They each obtained a mean *F*_1_-score of 0.64 across the experimental setups used for binary classification. The results also suggested that the final 12 to 16 weeks (about 3-4 months) of posts before a depressed user’s estimated diagnosis date are the most indicative of their illness, with data before that period not helping the models detect more accurately. Furthermore, in the 4- to 8-week period before the user’s estimated diagnosis date, their posts exhibited more negative sentiment than any other 4-week period in their post history.

**Conclusions:**

Transformer-based LMs may be used on data from web-based social media forums to identify users at risk for psychiatric conditions such as depression. Language features picked up by these classifiers might predate depression onset by weeks to months, enabling proactive mental health care interventions to support those at risk for this condition.

## Introduction

### Background

Major depressive disorder (MDD) is one of the most prevalent mental illnesses worldwide, affecting nearly 5% of adults [1]. Depressive episodes, which are symptoms of MDD and other psychiatric conditions, are even more common, with nearly 30% of individuals developing them at least once in their lifetime [2]. The characteristics of MDD and depressive episodes (“depression” hereafter) include low mood, feelings of worthlessness or guilt, and recurrent thoughts of death [3]. Early intervention has been reported to significantly improve patient outcomes and reduce the financial burden on health care services [4]. However, the stigma associated with psychiatric conditions, such as depression, leads to patients underreporting to health care services [5,6].

Given that a number of individuals who would normally meet the criteria for depression underreport to health care services, consideration should be given to how key symptoms may manifest in written language on social media platforms [7]. Longhand discussion websites such as Reddit are a rich source of such information where users may publish a series of posts spanning many months or years [8]. Natural language processing (NLP) can be used to identify features in posts that are predictive of a user who may have depression. Crucially, if affected users are identified before formal diagnosis, this may provide an opportunity for early health care intervention in these cases.

In this study, we derive a specialized subset of an annotated data set that contains Reddit posts belonging to users who have received a diagnosis of depression. This subset allowed us to consider posts before each user’s approximate diagnosis date.

We used state-of-the-art, domain-specific language models (LMs) to assist in the detection of depression. These LMs outperformed the baseline approaches in various experimental settings. Notably, they are adept at early detection of depression. Moreover, through our model analysis, we provide an exhaustive analysis of the temporal aspect related to preemptive detection, providing insights into the time depression symptoms materialized before the diagnosis. Finally, we investigated the role of sentiment in depressed users’ posts and provided a qualitative analysis based on the model performance.

### Related Work

There is a growing body of literature on the use of NLP techniques to analyze depression patterns on social media [9,10].

Yates et al [11] developed an approach to distinguish forum users who self-reported a diagnosis of depression from those who did not. It used a convolutional neural network to aggregate user posts in a purpose-built data set, the Reddit Self-reported Depression Diagnosis (RSDD) data set. Their follow-up work involved the conception of a sister data set, RSDD-Time [12], which contained Reddit posts where users declared a past diagnosis of depression, and this diagnosis was linked to an estimated date. Dates were inferred from explicit but often imprecise time expressions in user posts. However, these works did not consider the preemptive detection of depression among Reddit users in their data sets. That is, they did not consider methods for detecting depression in users before their diagnoses.

Recent NLP studies have explicitly focused on the early detection of depression. Preemptive detection of mentions of depression among Twitter users has been demonstrated with a degree of success by Owen et al [13]. Abed-Esfahani [14] reported similar findings using Reddit data. However, both studies were limited by the uncertainty of whether the users referring to this condition were formally diagnosed. Shah et al [15] also considered approaches for the early detection of depression in Reddit users. In this case, it was determined whether the user had received a physician’s diagnosis. However, it was not certain whether the users’ posts occurred before or after their diagnoses because the dates of the diagnoses were unknown. To gauge the effectiveness of the preemptive detection methods, a series of user posts before a known diagnosis date is required. Eichstaedt et al [16] examined the language in Facebook posts that may have been predictive of depression, as shown in patients’ medical records. They achieved an *F*_1_-score of 0.66 via logistic regression modeling, which used only the language preceding each patient’s depression diagnosis.

Therefore, this study also sought to extend existing work on preemptive depression detection. We considered social media users whose depression diagnosis date is known and used LMs to harness the language of user posts.

Ren et al [17] performed emotion-driven detection of depression using Reddit, achieving *F*_1_-scores exceeding 0.9. Their work considered individual depression posts, rather than a series of posts. Nevertheless, their effective use of emotional semantic information suggested that the dissection of our own results could be enhanced using sentiment analysis, which we included in our analysis to provide further insights.

### Objectives

We sought to gauge the performance of several machine learning classifiers in the task of distinguishing between RSDD data set users reporting and not reporting a diagnosis of depression, which from here onward we will term as “depressed” and “controls,” respectively. We then used the best-performing classifier in a temporally driven binary classification task. The purpose was to determine the volume of posts in a depressed user’s post timeline, which was the most indicative of their illness. To do this, we considered only the posts authored before the depressed users’ estimated diagnosis dates. Moreover, we considered only posts published up to 6 months before those dates.

The motivation for considering this 6-month time range hails from Winkour et al [18], and their observation that over 50% of patients with depression experienced their first onset at least 6 months before their formal diagnosis. Reece et al [19] made similar observations when examining Twitter users.

The time during which individuals with symptoms or traits of depression remain undiagnosed poses serious health risks. Patients who remain undiagnosed and thus untreated experience a worse outcome than would be the case if they were treated [20], particularly after their first episode [21]. Methods for assessing suitable time points for health care interventions are needed to identify ways to improve patient outcomes. They are also likely to advance the field of psychiatric therapeutics by supporting modifications to clinical guidelines or the design of randomized controlled trials [22]. A larger body of evidence on this matter could also help identify patients to be targeted for more thorough mental health assessments and provided with further resources, support, and treatment [23].

## Methods

### Data Description

#### Overview

Our work is based on the RSDD and RSDD-Time data sets [24]. The RSDD contains Reddit posts of 9210 depressed users and 108,731 control users. The posts were published between January 2006 and October 2016. The representation of users in RSDD is presented in Textbox 1.

RSDD-Time contains 598 annotated Reddit posts, each of which belongs to a user who declares that they have been formally diagnosed with depression. The posts were published between June 2009 and October 2016. Of these posts, 529 belonged to depressed users that were also present in the RSDD.

RSDD-Time annotations include the recency of a user’s diagnosis with respect to the date on which their post was authored. The permissible recency annotations are as follows:

0, unspecified; 1, in the past; 2, up to 2 months ago; 3, between 2 months and 1 year ago; 4, between 1 and 3 years ago; and 5, more than 3 years ago.

The representation of users in RSDD-Time is depicted in Textbox 2.

**An abstract representation of Reddit Self-reported Depression Diagnosis user data. It is not permissible to reveal true user IDs, post dates, or post texts due to privacy reasons.**

{user_id: 1, posts: [ (<date 1>, <text>),..., (<date n>, <text>) ], label: <either *depressed* or *control*>},

{user_id: 2, posts: [ (<date 1>, <text>),..., (<date n>, <text>) ], label: <either *depressed* or *control*>},

...,

{user_id: n, posts: [ (<date 1>, <text>),..., (<date n>, <text>) ], label: <either *depressed* or *control*>}

**An abstract representation of Reddit Self-reported Depression Diagnosis–Time user data. It is not permissible to reveal true user IDs, diagnosis post texts, or post dates, due to privacy reasons.**

{user_id: 1, diagnosis_post: <text>, post_date: <date>, recency: <*0*, *1*, *2*, *3*, *4*, or *5*>},

{user_id: 2, diagnosis_post: <text>, post_date: <date>, recency: <*0*, *1*, *2*, *3*, *4*, or *5*>},

...,

{user_id: n, diagnosis_post: <text>, post_date: <date>, recency: <*0*, *1*, *2*, *3*, *4*, or *5*>}

#### Deriving RSDD-Matched

We used this information to estimate the diagnosis dates of the 529 users present in both RSDD and RSDD-Time. Those with recency annotations of 0 or 1 were ignored because their diagnosis dates could not be estimated with any degree of accuracy. For each of the remaining users, we determined whether the estimated diagnosis date fell between the date of their first RSDD post and the date of their RSDD-Time diagnosis post. A total of 72 depressed users remained in the study.

A total of 10 matching control users were sought for each of the 72 depressed users. To accomplish this, candidate control users were randomly retrieved from the RSDD and analyzed sequentially. The candidates’ posts dated before the corresponding depressed user’s estimated diagnosis date were considered. If the number of posts belonging to the candidate did not vary by >15% with respect to the depressed user, the candidate was considered a match. A control user matched in this manner was not considered a candidate for subsequent depressed users.

Because sufficient matching control users could not be found for 2 of the depressed users, they were excluded from the resulting data set. The data set contained 70 depressed users, each of whom had 10 matching control users. Thus, there were a total of 770 users. The posts were published between April 2006 and June 2016. We named our data set RSDD-Matched. The characteristics of RSDD-Matched are shown in Table 1. Statistics pertaining to individual users in RSDD-Matched can be found in Multimedia Appendix 1.

Because RSDD does not include posts made in mental health subreddits, a depressed user’s diagnosis is certain to not be revealed until the time of their diagnosis post. There is language indicative of mental health conversation in the other subreddits.

**Table 1:** Statistics of the Reddit Self-reported Depression Diagnosis–Matched data set.

	Depressed users	Control users
Total users	70	700
Total posts	36,826	364,747
Total words	1,742,388	8,188,090
Average posts per user	526.1	521.1
Average words per post	47.3	22.4
Shortest post (words)	1	1
Longest post (words)	2642	1894

#### Descriptive Analysis of RSDD

To better understand our data set, we performed a simple descriptive analysis of RSDD. Word-level exploratory analyses of corpora have been extensively used in corpus linguistics and NLP to gain insight into word prominence. Typically, these follow a bag-of-words [25], pointwise mutual information [26], or term frequency–inverse document frequency (TF-IDF) [27] approach. In our case, we used lexical specificity [28], which is a statistical measure based on hypergeometric distribution, to identify the most prominent words in a corpus. We chose to use lexical specificity because it is structured in a way that is ideal for extracting corpus-specific vocabulary given a global corpus (RSDD) and its subsets (depressed and control users) [29]. It is also a more robust metric for term importance when dealing with different lengths of text [30], which is often the case for Reddit posts.

RSDD is partitioned into 2 subsets, or subcorpora, one containing posts of depressed users, and another containing posts of the control users. After lemmatizing the corpus, lexical specificity analysis revealed the unigrams (single words) that were the most frequently used by depressed and control participants (Table 2). The score column indicates the relevance of a unigram to each subset. For reference, the term “woman” makes up 0.18% (460,893/257,873,124) of the total words that appear in the depressed user subset compared with only 0.06% (569,330/950,988,726) of the control user subset.

To put the results into context, we should mention that a lexical specificity score of X for a given word W with frequency f means that the probability of W occurring at least f times in the subcorpus is lower than 10^–X^ (assuming a random distribution). For instance, a lexical specificity score of 42,234 for “game” means that the probability of “game” having a frequency of f=5,373,938 or higher in the control users subcorpus is 10^–42,234^ (ie, an exceptionally low probability which means “game” is overrepresented in the control users’ subset). In general, we can observe a pattern in which depressed users tend to use more relationship or family-related words (eg, “woman” or “relationship”) and words related to the depression symptoms themselves (eg, “life”). In contrast, control users seem to use more mundane terms related to the subreddit communities, such as game-related terms (eg, “game” or “team”). Although this analysis is based only on the statistical frequency of the terms used, it may provide further evidence that developing automatic methods to identify users with depression may indeed be feasible. In the *Results* section, we extend this initial inspection to better understand the errors made by the automatic models.

**Table 2:** Top ranked words of Reddit Self-reported Depression Diagnosis depressed and control users in terms of lexical specificity.

User, word			Score
**Depressed users**			
	people	338,131.45	
	know	164,368.51	
	thing	150,440.49	
	feel	118,483.23	
	time	97,250.09	
	woman	96,165.35	
	go	79,611.79	
	want	75,379.17	
	life	67,769.01	
	relationship	62,606.64	
**Control users**			
	game	42,234.94	
	trade	39,445.65	
	key	30,031.17	
	team	24,333.73	
	play	17,389.38	
	player	16,186.61	
	shiny	14,032.27	
	hatch	13,265.87	
	thank	10,177.49	
	add	10,005.14	

### Methodology

In this section, we provide more details of our proposed methods for tackling the depression detection task. Framing the task as a machine learning problem, we considered 9 methods based on linear classifiers and more recent LMs.

The initial baselines entailed a support vector machine (SVM) architecture. SVM is an algorithm that learns by example to assign labels to objects [31]. In our case, the objects are Reddit users, and permissible labels are “depressed” and “control.” SVMs have demonstrated effectiveness in the detection of depression-related posts in Reddit [8,32]. Our SVM configurations used different features derived from user posts. These features included TF-IDF, word embeddings, and a combination of both TF-IDF and word embeddings. The TF-IDF [33] features represent the words deemed most notable among the user posts. Word embedding is a real-valued vector representation of a word [34]. Words with similar meanings have vectors with similar values.

The SVM model used was that of scikit-learn [35], as was the TF-IDF vectorizer implementation. The word embeddings generated for each Reddit post were drawn from global vectors trained on Wikipedia and Gigaword data [36]. These vectors had a dimensionality of 300, similar to the average embedding generated. We performed Reddit posttext preprocessing before their input to the SVM. All posts underwent quotation normalization; therefore, each quotation character was represented by a single apostrophe. All new lines and carriage return characters were replaced with spaces so that posts were represented as a single line string. The posts were then concatenated on a per-user basis so that each user’s posting history was represented as a single-line string. SVM used a linear kernel, which is appropriate for text-classification problems [37-39].

The remaining 6 classifiers were transformer-based LMs. LMs are a statistical means of predicting words [40], whereas transformers provide a neural-network-based approach to generating such models [41]. Transformer-based LMs have proven effective in detecting psychiatric illness-related Reddit posts [12,42,43]. Therefore, we chose to use transformer-based LMs to support the detection of depression in RSDD-Matched. We chose Bidirectional Encoder Representations from Transformers (BERT) [44] and A Lite BERT (ALBERT) [45], which are appropriate for a wide variety of applications. We also chose 4 specialist LMs: BioBERT [46], Longformer [47], MentalBERT [48], and MentalRoBERTa [48]. BioBERT is suitable for use where biomedical concepts are prevalent, such as electronic medical records [49], patient descriptions [50], and health-related Twitter posts [51]. Longformer is designed for use when text is formed from long documents. Indeed, there were posts in RSDD-Matched that exceed 2000 words. Finally, MentalBERT and MentalRoBERTa are customized for the domain of mental health care and trained using text drawn from mental health discussion forums.

All 6 transformer-based LMs were pretrained bidirectional language representations. This means that for any given word in a text segment, its neighboring words to both the left and right are examined so that the context of the word is well understood. These representations lend themselves to high performance in text classification tasks when compared with traditional approaches using SVMs, for example [52,53].

We used the Simple Transformers software library [54] to deploy LMs. The library provides an application programming interface to the transformer library, which itself provides access to the BERT, ALBERT, BioBERT, Longformer, MentalBERT, and MentalRoBERTa models [55]. The BERT, ALBERT, BioBERT, Longformer, MentalBERT, and MentalRoBERTa classifiers used were “bert-base-uncased,” “albert-base-v1,” “biobert-base-cased-v1.1,” “longformer-base-4096,” “mental-bert-base-uncased,” and “mental-roberta-base,” respectively. In addition to the default hyperparameters of the Simple Transformers, the LM classifiers were instantiated, with the sliding window enabled. Transformer-based LMs may consume only a limited number of tokens (512 tokens). Because the posting histories of most users in RSDD-Matched exceed 512 words, a specialist approach to applying LMs to these posts is needed. Sliding window is one such approach [56].

### Experimental Setup

#### Preemptive Depression Identification Experiment

The first experiment examined the performance of several machine learning classifiers in the task of distinguishing between depressed and control users in RSDD-Matched. The purpose of this experiment was to understand the extent to which the preemptive detection of depression in social media is possible. Moreover, this experiment was aimed at understanding the capabilities of machine learning classifiers for this task and the suitability of different methods in the task. The results were used to provide a competitive model for subsequent fine-grained temporal experiments.

We used 9 different classifiers. Three entailed an SVM, as described in the *Methodology* section. The remaining 6 were BERT, ALBERT, BioBERT, Longformer, MentalBERT, and MentalRoBERTa, which are also described in the *Methods* section.

In addition to the aforementioned classifiers, we included a naive baseline that predicted positive instances in all cases.

Because the number of positive instances (ie, depressed users) in RSDD-Matched was small, we chose not to use a traditional train-test split. Instead, we used 5-fold cross-validation; an approach also used by Eichstaedt et al [14]. Furthermore, we varied the number of matching control users across the 4 iterations of the experiment (Table 3).

The purpose of these variations is to test the performance of classifiers against increasingly imbalanced data sets. This mimics the conditions likely to be observed in web-based forums where the number of positive instances (ie, depressed users) is dwarfed by the number of negative instances (ie, nondepressed users).

**Table 3:** Variations of the preemptive depression identification experiment in terms of the number of matching control users considered.

	Depressed users	Matching control users per depressed user	Total users
Variation 1	70	1	140
Variation 2	70	3	280
Variation 3	70	5	420
Variation 4	70	10	770

#### Temporal Experiment

The purpose of the second primary experiment was to determine which posting period in a depressed user’s post timeline was the most indicative of depression. This involved the use of a subset of RSDD-Matched users. The performance of binary classifiers versus temporal subsets of the posts in the 6 months before the users’ estimated diagnosis dates was measured.

The RSDD-Matched subset contained only depressed users who had at least one post in the 2 weeks before their estimated diagnosis date. Of the 70 depressed users in our RSDD subset, 14 did not have any posts in this 2-week period. Consequently, we used only 56 depressed users in the temporal experiment. Furthermore, not all 10 control users matched with each of the 56 depressed were useable because some did not have at least one post in this 2-week period. Thus, we performed additional random exclusions of controls to rebalance the data set. After these exclusions, the data set used in the temporal experiment contained 56 depressed users, each of which had 3 matching control users, totaling to 224 users.

The results of the preemptive depression identification experiment were used to partially inform the design of the temporal experiment. Because BERT scored the highest average *F*_1_-score across all runs of the preemptive depression identification experiment, it was decided that this was the sole general-purpose transformer-based LM to be used in the temporal experiment. Likewise, MentalBERT had the highest average *F*_1_-score; therefore, it was selected as the sole specialist LM. The 3 variations of the SVM classifier used in the preemptive depression-identification experiment were used once again.

Once again, we used 5-fold cross-validation. Two chief variations of the RSDD-Matched subset and several different temporal configurations were used (Table 4).

**Figure 1:**
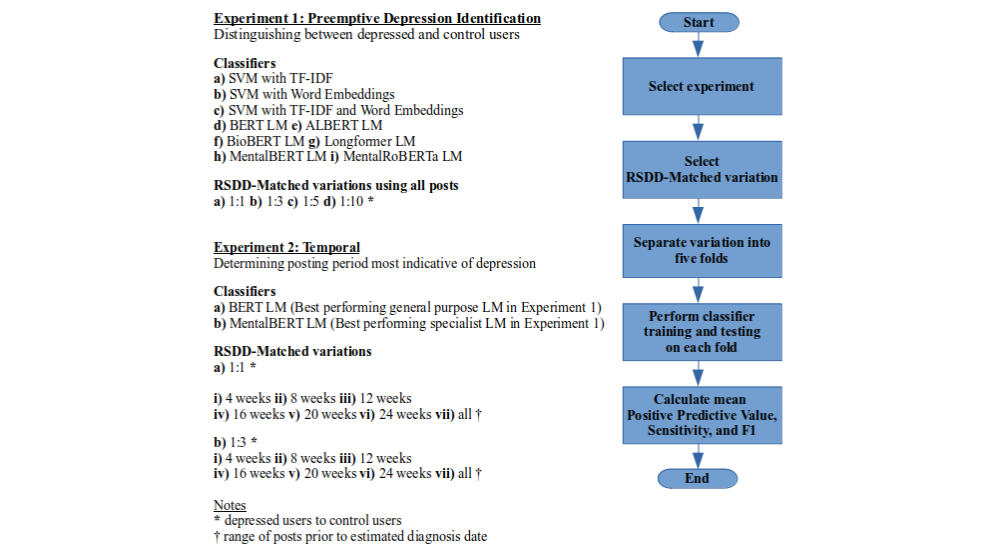
Summary of the 2 chief experimental setups. ALBERT: A Lite Bidirectional Encoder Representations from Transformers; BERT: Bidirectional Encoder Representations from Transformers; LM: language model; SVM: support vector machine; TF-IDF: term frequency–inverse document frequency; RSDD: Reddit Self-reported Depression Diagnosis.

The 2 chief strands to our experimental setup are summarized in Figure 1.

We complemented the temporal experiment with sentiment analysis. The purpose of this study was to identify whether there is a link between sentiment and depression with respect to user posts. Text sentiment has been extensively used as a predictor for detecting signs of depressive mood in microblog users [57-59]. Specifically, negatively charged text has often been correlated with depression via expressions of low mood and suicidal ideation [60]. Approaches used to extract sentiment from social media posts include the use of LMs [61] and lexicons such as Valence Aware Dictionary and Sentiment Reasoner (VADER) [62].

To determine whether there is a relationship between sentiment and depression, we used BERTweet-sentiment, a state-of-the-art transformer model, to classify each post in RSDD-Matched as either negative, neutral, or positive. BERTweet-sentiment is based on the BERTweet [63] implementation, which is trained on a large Twitter corpus and fine-tuned for sentiment analysis. Although the model is not trained on Reddit data, we believe that there are enough overlapping lexical characteristics between the 2 domains in terms of internet slang and text lengths that justify its use.

Our sentiment analysis focused on changes in the sentiment distribution of depressed and control users over time. In step with the design of our temporal experiment, each user’s posts are divided into 6 temporal bands, namely 0-4, 4-8, 8-12, 12-16, 16-20, and 20-24 weeks before their estimated diagnosis date (for a control user, this is the estimated diagnosis of its matched depressed user). The average percentage of each sentiment in each band was considered.

To establish whether the diagnosis was associated with the sentiment of a post, 2 regression models were used. The first was based on the *lme4* framework [64], and the second on *mgcv* [65]. The implementations used were those of the R (version 4.02) statistical environment [66]. We set our outcome variable to be whether a post is “sentimental” (that is, either negative or positive) or not (neutral), and a logistic mixed effects regression was fitted using all the available posts with the individual user identifier as a random effect term. As fixed effects, we used the estimated depression diagnosis (ie, either depressed or control), the time to estimated diagnosis in weeks, the post’s word count, and the interaction term of estimated diagnosis with time.

Having sought to establish whether the diagnosis of the user was associated with the sentimentality inferred for each post, we also considered a more fine-grained multinomial regression model. This is equivalent to fitting a series of logistic models against a reference category [67] and is similar to the “stacked” designs used in other disciplines [68]. For our purposes, we will consider “neutral” as the reference category of our multinomial outcome, so all effect sizes will indicate the probability of a post being positive or negative *instead of* neutral.

**Table 4:** Variations of the temporal experiment in terms of the number of matching control users and numbers of weeks of posts before estimated diagnosis dates considered.

	Depressed users	Matching control users per depressed user	Total users	Weeks of posts included before estimated diagnosis date
Variation 1	56	1	112	4, 8, 12, 16, 20, and 24
Variation 2	56	3	224	4, 8, 12, 16, 20, and 24

## Results

### Preemptive Depression Identification Experiment

The results of the preemptive depression identification experiment are presented in Tables 5-8. Each table shows a variation in the number of matched control users. Positive predictive value, sensitivity, and *F*_1_-score were used to measure the performance in each variation. The positive predictive value denotes the number of users classified as depressed who were indeed depressed. Sensitivity denotes how many of the depressed users were correctly classified as depressed. The *F*_1_-score, which is the harmonic mean of the positive predictive value and sensitivity, is suitable for use with data sets such as ours, where the class distribution (of depressed and controls) is uneven [69]. In contrast, accuracy is not suitable for such data sets [70]. Therefore, we used *F*_1_-score as the primary performance metric.

Using *F*_1_-score as a primary performance indicator, MentalBERT performs best across the variations.

A detailed breakdown of the results of the preemptive depression identification experiment can be found in Multimedia Appendix 1.

Word embeddings (vector representations) result in strong sensitivity (recall), whereas TF-IDF features cause deficient performance. The positive predictive value (precision) was best observed when using the specialist LM, MentalBERT. The best *F*_1_-score was also achieved by MentalBERT and exceeded the naive baseline.

We now consider the selected users from RSDD-Matched and the performance of the classifiers against them. We will examine one misclassified user per variation in the experiment (in terms of depressed users and the number of matched controls). For each variation, we will examine the strongest performing classifier and the user that it misclassified with the highest probability.

To identify the potential reasons for the misclassifications, we examined the lexical properties of user posts using 3 approaches. The first approach involves ascertaining the chief topic conveyed by the posts, a topic represented by 5 words. Topic modeling via latent Dirichlet allocation was used to accomplish this [71,72]. The second approach examines the chief TF-IDF features of the user posts. The third approach is to count the frequencies of depressed and control vocabularies (Table 2) that appear across the posts.

We present the misclassified depressed users with respect to each variation in the experiment (Table 9). We also present the misclassified control users with respect to each variation (Table 10).

One depressed user is often misclassified. User d13 was deemed a control user using 3 different classifiers across 3 different variations. Although depressed vocabulary counts slightly outweigh their control counterparts, the totals for both vocabularies were nominal. The topic of the user’s posts is probably more indicative of the reasons for the misclassification. Certainly, a theme concerning death or dying appears to be present, but this is diluted by optimistic sounding references of temporal and geographic nature. Further diluting references are revealed among the TF-IDF features, where strong terms such as “love” are present. It seems that the classifiers construe such references as those belonging to a control user.

User d38 may have been misclassified for similar reasons. Counts for both depressed and control vocabularies were small. Positive terms, such as “welcome” and “invite” might be deemed to belong to a control user.

An inferior performance was observed across the classifiers in the most imbalanced environment. We examine depressed user d57, which has been misclassified with a probability close to certainty. The depressed vocabulary count dwarfs the control vocabulary count. However, when making its decision, the classifier seems to harness the overarching nature of the user’s posts, as indicated by the topic model and TF-IDF features. The prevalence of “good” natured posts will inevitably see the user deemed similar to a control user when represented in a vector space.

We now consider misclassified control users with respect to each variation in the experiment (Table 10).

Certain users appear to be confounding across several different classifiers and variations. User c13 was strongly misclassified as a depressed user by both MentalBERT and MentalRoBERTa in the relatively noisy environments of 3 and 5 matched control users, respectively (Table 10). The depressed vocabulary counts far outweigh the control vocabulary counts for this user. In addition, the theological topic and TF-IDF features of the user’s posts are deemed likely to be those of a depressed user, according to the classifier.

MentalBERT demonstrated adeptness in the most balanced variation in the experiment. We sought possible explanations for the misclassification of user c521. The control vocabulary count slightly outweighed that of depressed vocabulary. Moreover, the topic model and TF-IDF features are composed of terms that complement the control vocabulary. Intuitive reasons for misclassification as depressed are difficult to cite. Therefore, it is possible that, in a balanced environment, the classifier simply has too few control users to compare with depressed users.

In the noisiest environment, the simpler word-based model (SVM using word embeddings) demonstrated the strongest performance. Transformer-based language modeling cannot be performed. The vocabulary of the most strongly misclassified user in this case (c535) only offers a tenuous explanation. The count of depressed vocabulary was small, although it outweighed that of the control vocabulary. However, the topic and TF-IDF terms appeared to complement the depressed vocabulary, which may have been the cause of the misclassification.

**Table 5:** Binary classification scores using all posts of 70 depressed users and 1 of their matched control users^a^.

	Positive predictive value, mean (SD)	Sensitivity, mean (SD)	*F*_1_-score, mean (SD)
SVM^b^ using TF-IDF^c^	0.637 (N/A^d^)	0.557 (N/A)	0.590 (N/A)
SVM using word embeddings	0.558 (N/A)	0.543 (N/A)	0.548 (N/A)
SVM using TF-IDF and word embeddings	0.673 (N/A)	0.557 (N/A)	0.596 (N/A)
BERT^e^ LM^f^	0.638 (0.021)	0.805 (0.022)	0.709 (0.012)
ALBERT^g^ LM	0.606 (0.008)	0.786 (0.015)	0.683 (0.010)
BioBERT LM	0.601 (0.005)	0.862 (0.022)	0.707 (0.005)
Longformer LM	0.633 (0.009)	0.838 (0.036)	0.719 (0.018)
MentalBERT LM	0.660 (0.019)	0.848 (0.008)	0.738 (0.013)
MentalRoBERTa LM	0.629 (0.002)	0.819 (0.022)	0.709 (0.006)
Naive baseline—all depression	0.500 (N/A)	1.000 (N/A)	0.667 (N/A)

^a^Language model experiments were run 3 times each, therefore both mean and SD scores are provided.

^b^SVM: support vector machine.

^c^TF-IDF: term frequency–inverse document frequency.

^d^N/A: not applicable.

^e^BERT: Bidirectional Encoder Representations from Transformers.

^f^LM: language model.

^g^ALBERT: A Lite Bidirectional Encoder Representations from Transformers.

**Table 6:** Binary classification scores using all posts of 70 depressed users and 3 of their matched control users^a^.

	Positive predictive value, mean (SD)	Sensitivity, mean (SD)	*F*_1_-score, mean (SD)
SVM^b^ using TF-IDF^c^	0.800 (N/A^d^)	0.086 (N/A)	0.153 (N/A)
SVM using word embeddings	0.411 (N/A)	0.529 (N/A)	0.459 (N/A)
SVM using TF-IDF and word embeddings	0.800 (N/A)	0.057 (N/A)	0.107 (N/A)
BERT^e^ LM^f^	0.653 (0.033)	0.481 (0.022)	0.546 (0.025)
ALBERT^g^ LM	0.652 (0.034)	0.476 (0.009)	0.547 (0.018)
BioBERT LM	0.654 (0.028)	0.410 (0.030)	0.496 (0.020)
Longformer LM	0.653 (0.036)	0.476 (0.036)	0.534 (0.031)
MentalBERT LM	0.657 (0.034)	0.509 (0.008)	0.562 (0.016)
MentalRoBERTa LM	0.614 (0.023)	0.471 (0.015)	0.522 (0.002)
Naive baseline—all depression	0.250 (N/A)	1.000 (N/A)	0.167 (N/A)

**^a^**Language model experiments were run 3 times each, therefore both mean and SD scores are provided.

^b^SVM: support vector machine.

^c^TF-IDF: term frequency–inverse document frequency.

^d^N/A: not applicable.

^e^BERT: Bidirectional Encoder Representations from Transformers.

^f^LM: language model.

^g^ALBERT: A Lite Bidirectional Encoder Representations from Transformers.

**Table 7:** Binary classification scores using all posts of 70 depressed users and 5 of their matched control users^a^.

	Positive predictive value, mean (SD)	Sensitivity, mean (SD)	*F*_1_-score, mean (SD)
SVM^b^ using TF-IDF^c^	0.400 (N/A^d^)	0.029 (N/A)	0.053 (N/A)
SVM using word embeddings	0.309 (N/A)	0.471 (N/A)	0.372 (N/A)
SVM using TF-IDF and word embeddings	0.200 (N/A)	0.014 (N/A)	0.027 (N/A)
BERT^e^ LM^f^	0.615 (0.028)	0.290 (0.022)	0.379 (0.017)
ALBERT^g^ LM	0.555 (0.030)	0.281 (0.009)	0.354 (0.006)
BioBERT LM	0.627 (0.034)	0.252 (0.021)	0.331 (0.027)
Longformer LM	0.624 (0.108)	0.286 (0.038)	0.363 (0.059)
MentalBERT LM	0.572 (0.002)	0.329 (0.043)	0.400 (0.040)
MentalRoBERTa LM	0.562 (0.027)	0.343 (0.000)	0.419 (0.010)
Naive baseline—all depression	0.167 (N/A)	1.000 (N/A)	0.286 (N/A)

^a^Language model experiments were run 3 times each, therefore both mean and SD scores are provided.

^b^SVM: support vector machine.

^c^TF-IDF: term frequency–inverse document frequency.

^d^N/A: not applicable.

^e^BERT: Bidirectional Encoder Representations from Transformers.

^f^LM: language model.

^g^ALBERT: A Lite Bidirectional Encoder Representations from Transformers.

**Table 8:** Binary classification scores using all posts of 70 depressed users and 10 of their matched control users^a^.

	Positive predictive value, mean (SD)	Sensitivity, mean (SD)	*F*_1_-score, mean (SD)
SVM^b^ using TF-IDF^c^	0.000 (N/A^d^)	0.000 (N/A)	0.000 (N/A)
SVM using word embeddings	0.212 (N/A)	0.371 (N/A)	0.268 (N/A)
SVM using TF-IDF and word embeddings	0.000 (N/A)	0.000 (N/A)	0.000 (N/A)
BERT^e^ LM^f^	0.100 (0.000)	0.014 (0.000)	0.025 (0.00)
ALBERT^g^ LM	0.089 (0.019)	0.014 (0.000)	0.025 (0.001)
BioBERT LM	0.067 (0.115)	0.005 (0.008)	0.009 (0.016)
Longformer LM	0.024 (0.019)	0.019 (0.033)	0.021 (0.037)
MentalBERT LM	0.167 (0.058)	0.014 (0.000)	0.026 (0.001)
MentalRoBERTa LM	0.272 (0.185)	0.034 (0.008)	0.057 (0.018)
Naive baseline—all depression	0.091 (N/A)	1.000 (N/A)	0.167 (N/A)

^a^Language model experiments were run 3 times each, therefore both mean and SD scores are provided.

^b^SVM: support vector machine.

^c^TF-IDF: term frequency–inverse document frequency.

^d^N/A: not applicable.

^e^BERT: Bidirectional Encoder Representations from Transformers.

^f^LM: language model.

^g^ALBERT: A Lite Bidirectional Encoder Representations from Transformers.

**Table 9:** Depressed users most strongly misclassified in each variation of the preemptive depression identification experiment^a^.

		One depression user per control user (1:1)	One depression user per 3 control users (1:3)	One depression user per 5 control users (1:5)	One depression user per 10 control users (1:10)
Classifier		MentalBERT LM^b^	MentalBERT LM	MentalRoBERTa LM	SVM^c^ using word embeddings
User		d13	d38	d13	d57
Control probability		0.93	0.94	0.99	0.98
Sum of post lengths in words		1696	1888	1696	55,897
Topic		news hawaii time dead blue	sir-geo welcomed invite leave warlock	news hawaii time dead blue	good time people years problem
Chief TF-IDF^d^ features		love minnesota diablo time man bud zoidberg like month hawaii	sir geo welcome invite warlock leave titan psn run need	love minnesota diablo time man bud zoidberg like month hawaii	good know use make time thank link want try like
**Depressed vocabulary counts**					
	people	1	1	1	64
	know	6	0	6	93
	thing	3	0	3	35
	feel	2	2	2	10
	time	5	8	5	99
	woman	1	0	1	7
	go	3	0	3	54
	want	3	1	3	71
	life	2	0	2	28
	relationship	0	0	0	2
**Control vocabulary counts**					
	game	0	1	0	9
	trade	0	0	0	2
	key	0	0	0	4
	team	2	3	2	4
	play	0	1	0	35
	player	0	0	0	8
	shiny	0	0	0	0
	hatch	0	0	0	0
	thank	1	1	0	15
	add	0	2	0	14

^a^Lexical properties of those users’ posts are provided.

^b^LM: language model.

^c^SVM: support vector machine.

^d^TF-IDF: term frequency–inverse document frequency.

**Table 10:** Control users most strongly misclassified in each variation of the preemptive depression identification experiment^a^.

		One depression user per control user (1:1)	One depression user per 3 control users (1:3)	One depression user per 5 control users (1:5)	One depression user per 10 control users (1:10)
Classifier		MentalBERT LM^b^	MentalBERT LM	MentalRoBERTa LM	SVM^c^ using Word embeddings
User		c521	c13	c13	c535
Depressed probability		0.99	0.95	0.91	0.91
Sum of post lengths in words		1513	8489	8489	1595
Topic		elo play team bronze games	god jesus people good life	god jesus people good life	people shit reddit guy man
Chief TF-IDF^d^ features		team just suck elo play game like good sydtko win	god think way thing try know jesus people say like	god think way thing try know jesus people say like	say thank guy people reddit man make tell watch let
**Depressed vocabulary counts**					
	people	4	48	48	6
	know	2	36	36	3
	thing	3	28	28	1
	feel	1	6	6	1
	time	2	6	6	4
	woman	0	4	4	0
	go	0	4	4	5
	want	3	16	16	1
	life	0	46	46	1
	relationship	0	8	8	0
**Control vocabulary counts**					
	game	7	0	0	0
	trade	0	0	0	0
	key	0	0	0	0
	team	9	0	0	0
	play	9	6	6	0
	player	2	0	0	0
	shiny	0	0	0	0
	hatch	0	0	0	0
	thank	1	4	4	1
	add	1	0	0	0

^a^Lexical properties of those users’ posts are provided.

^b^LM: language model.

^c^SVM: Support Vector Machine.

^d^TF-IDF: Term Frequency–-Inverse Document Frequency.

### Temporal Experiment

**Figure 2:**
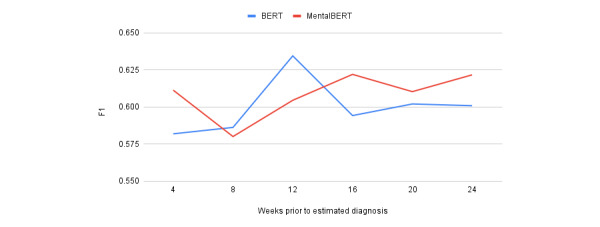
Average performances of Bidirectional Encoder Representations from Transformers (BERT) and MentalBERT between 4 and 24 weeks before the estimated diagnosis date.

We then performed a temporal experiment. Because BERT achieved the highest *F*_1_-score across all preemptive depression identification experiment variations, it was selected as the exclusive general-purpose LM here. For the same reason, MentalBERT was selected as an exclusive specialist LM. The results are presented in Tables 11 and 12. Each table shows a variation in the number of matched control users. The average performance of each LM across the 2 variations is shown in Figure 2.

For BERT, the strongest sensitivity and *F*_1_-scores were observed when only 12 weeks (approximately 3 months) of posts before the estimated diagnosis dates were considered. Subsets larger or smaller than 12 weeks caused degradation in the classifier performance. For MentalBERT, the strongest sensitivity and *F*_1_-scores were obtained when either 16 or 24 weeks of posts were considered. With BERT scoring a higher *F*_1_-score at 12 weeks than MentalBERT, this suggests that the final 12 weeks of posts before a depressed user’s estimated diagnosis date may be the most indicative of their illness.

An explanation for the slightly inferior performance of MentalBERT may be found in its construction: it is pretrained on text from mental health subreddits such as “r/depression” and “r/mental health” [48]. However, RSDD (from which we derived RSDD-Matched) does not contain posts from mental health subreddits. Therefore, when RSDD-Matched data are limited, as in our temporal experiment, more general-purpose models, such as BERT, may be able to achieve stronger performance. BERT is pretrained on more general corpora, such as Wikipedia [44].

A detailed breakdown of the results of the temporal experiment can be found in Multimedia Appendix 1.

We once again consider selected users from RSDD-Matched and the performance of the classifiers against them. We again examined one misclassified user per variation in the experiment (in terms of depressed users and number of matched controls). For each variation, we will examine the strongest performing time span, and the user that is misclassified with the highest probability. To identify the reasons for the misclassifications, we again examined the lexical properties of the user posts using topic models, TF-IDF features, and vocabulary (Table 2) frequency counts.

Misclassified depressed users with respect to the 2 variations in the experiment are listed in Table 13.

User d52 is a depressed user misclassified in both balanced and imbalanced environments, where only the final 12 weeks of their posts are considered. The vocabulary of these posts intersected with very little of the chief depressed vocabulary. It intersects with slightly more of the chief control vocabulary. The topic and TF-IDF features, intuitively speaking, appear to belong to that of a control rather than a depressed user. Perhaps, a balanced environment with temporally limited post histories provides little training data from which the classifier can learn to differentiate between controls and depressed users. Although rare, these cases may occur in practice and highlight the importance of being careful in overrelying on automatic models for individual assessments without human expert intervention.

We now consider the misclassified control users with respect to the 2 variations in the experiment (Table 14).

First, we consider user c481. Both its depressed and control vocabulary counts were zero, which offers some insight into misclassification. The topic and TF-IDF features of the posts appear to align with those of the control user. However, it is likely that the prevalence of “pain” is a confounding factor. This term may be intuitively linked to depressed users, which may mislead the classifier. Again, the limited temporal range of posts in this setting provided little data from which the classifier could learn.

User c13 is a confounder in the preemptive depression identification experiment and has been proven to be so in the temporal experiment. Even when considering only the last 12 weeks of the user’s posts in an imbalanced environment, theologically themed vocabulary is not diluted. It intersects strongly with the vocabulary of depressed users and explains this misclassification.

**Table 11:** Binary classification scores using 56 depressed users and 1 of their matched control users and 6 temporal post subsets^a^.

			Positive predictive value, mean (SD)		Sensitivity, mean (SD)		*F*_1_-score, mean (SD)
**Last 4 weeks**							
	BERT^b^ LM^c^	0.575 (0.027)		0.830 (0.039)		0.675 (0.023)	
	MentalBERT LM	0.612 (0.026)		0.835 (0.026)		0.698 (0.017)	
**Last 8 weeks**							
	BERT LM	0.598 (0.026)		0.854 (0.071)		0.700 (0.037)	
	MentalBERT LM	0.603 (0.020)		0.842 (0.047)		0.699 (0.022)	
**Last 12 weeks**							
	BERT LM	0.605 (0.014)		0.912 (0.018)		0.726 (0.015)	
	MentalBERT LM	0.600 (0.013)		0.888 (0.010)		0.715 (0.008)	
**Last 16 weeks**							
	BERT LM	0.570 (0.009)		0.863 (0.026)		0.684 (0.007)	
	MentalBERT LM	0.575 (0.009)		0.907 (0.028)		0.703 (0.016)	
**Last 20 weeks**							
	BERT LM	0.569 (0.023)		0.893 (0.036)		0.694 (0.025)	
	MentalBERT LM	0.578 (0.018)		0.882 (0.027)		0.696 (0.014)	
**Last 24 weeks**							
	BERT LM	0.565 (0.021)		0.871 (0.027)		0.683 (0.010)	
	MentalBERT LM	0.591 (0.014)		0.890 (0.010)		0.707 (0.011)	
**All posts**							
	BERT LM	0.627 (0.018)		0.824 (0.032)		0.710 (0.019)	
	MentalBERT LM	0.638 (0.009)		0.861 (0.000)		0.732 (0.006)	
Naive baseline			0.500 (N/A^d^)		1.000 (N/A)		0.667 (N/A)

**^a^**The classifiers used are BERT LM and MentalBERT LM, both of whose experiments were run 3 times each, therefore both mean and SD scores are provided.

^b^BERT: Bidirectional Encoder Representations From Transformers.

^c^LM: language model.

^d^N/A: not applicable.

**Table 12:** Binary classification scores using 56 depressed users and 3 of their matched control users and 6 temporal post subsets^a^.

			Positive predictive value, mean (SD)		Sensitivity, mean (SD)		*F*_1_-score, mean (SD)
**Last 4 weeks**							
	BERT^b^ LM^c^	0.480 (0.027)		0.538 (0.019)		0.489 (0.010)	
	MentalBERT LM	0.494 (0.019)		0.577 (0.009)		0.525 (0.007)	
**Last 8 weeks**							
	BERT LM	0.446 (0.032)		0.538 (0.036)		0.472 (0.035)	
	MentalBERT LM	0.427 (0.027)		0.524 (0.029)		0.461 (0.023)	
**Last 12 weeks**							
	BERT LM	0.498 (0.031)		0.619 (0.037)		0.543 (0.035)	
	MentalBERT LM	0.448 (0.007)		0.569 (0.017)		0.494 (0.009)	
**Last 16 weeks**							
	BERT LM	0.471 (0.010)		0.565 (0.021)		0.504 (0.011)	
	MentalBERT LM	0.481 (0.023)		0.643 (0.037)		0.541 (0.028)	
**Last 20 weeks**							
	BERT LM	0.475 (0.039)		0.577 (0.037)		0.510 (0.034)	
	MentalBERT LM	0.487 (0.018)		0.595 (0.011)		0.524 (0.009)	
**Last 24 weeks**							
	BERT LM	0.470 (0.033)		0.591 (0.036)		0.518 (0.033)	
	MentalBERT LM	0.501 (0.022)		0.591 (0.018)		0.536 (0.022)	
**All posts**							
	BERT LM	0.625 (0.021)		0.519 (0.032)		0.562 (0.015)	
	MentalBERT LM	0.588 (0.005)		0.508 (0.010)		0.540 (0.003)	
Naive baseline			0.250 (N/A^d^)		1.000 (N/A)		0.400 (N/A)

^a^The classifiers used are BERT LM and MentalBERT LM, both of whose experiments were run 3 times each, therefore both mean and SD scores are provided..

^b^BERT: Bidirectional Encoder Representations From Transformer.

^c^LM: language model.

^d^N/A: not applicable.

**Table 13:** Depressed users most strongly misclassified in each variation of the temporal experiment. Lexical properties of those users’ posts are provided.

		One depression user per control user (1:1)	One depression user per 3 control users (1:3)
Time span		Last 12 weeks	Last 12 weeks
Classifier		BERT^a^ LM^b^	BERT LM
User		d52	d52
Control probability		0.869	0.935
Sum of post lengths in words		1225	1225
Topic		england belgium hamster time team	england belgium hamster time team
Chief TF-IDF^c^ features		thank team player help time goal cage post second start	thank team player help time goal cage post second start
**Depressed vocabulary counts**			
	people	0	0
	know	1	1
	thing	1	1
	feel	0	0
	time	4	4
	woman	0	0
	go	0	0
	want	2	2
	life	0	0
	relationship	0	0
**Control vocabulary counts**			
	game	2	2
	trade	0	0
	key	0	0
	team	4	4
	play	0	0
	player	1	1
	shiny	0	0
	hatch	0	0
	thank	2	2
	add	1	1

^a^BERT: Bidirectional Encoder Representations From Transformers.

^b^LM: language model.

^c^TF-IDF: term frequency–inverse document frequency.

**Table 14:** Control users most strongly misclassified in each variation of the temporal experiment. Lexical properties of those users’ posts are provided.

		One depression user per control user (1:1)	One depression user per 3 control users (1:3)		
Time span		Last 12 weeks	Last 12 weeks		
Classifier		BERT^a^ LM^b^	BERT LM		
User		c481	c13		
Depressed probability		0.963	0.917		
Total length of posts in words		258	8489		
Topic		food clove tomorrow pain suspect	god jesus people good life		
Chief TF-IDF^c^ features		reply eat food cat clove pain suspect tooth vet water	god think way thing try know jesus people say like		
**Depressed vocabulary counts**					
	people	0	24		
	know	0	18		
	thing	0	14		
	feel	0	3		
	time	0	3		
	woman	0	2		
	go	0	2		
	want	0	8		
	life	0	23		
	relationship	0	4		
**Control vocabulary counts**					
	game	0	0		
	trade	0	0		
	key	0	0		
	team	0	0		
	play	0	3		
	player	0	0		
	shiny	0	0		
	hatch	0	0		
	thank	0	2		
	add	0	0		

^a^BERT: Bidirectional Encoder Representations From Transformers.

^b^LM: language model.

^c^TF-IDF: term frequency–inverse document frequency.

### Sentiment Analysis

**Figure 3:**
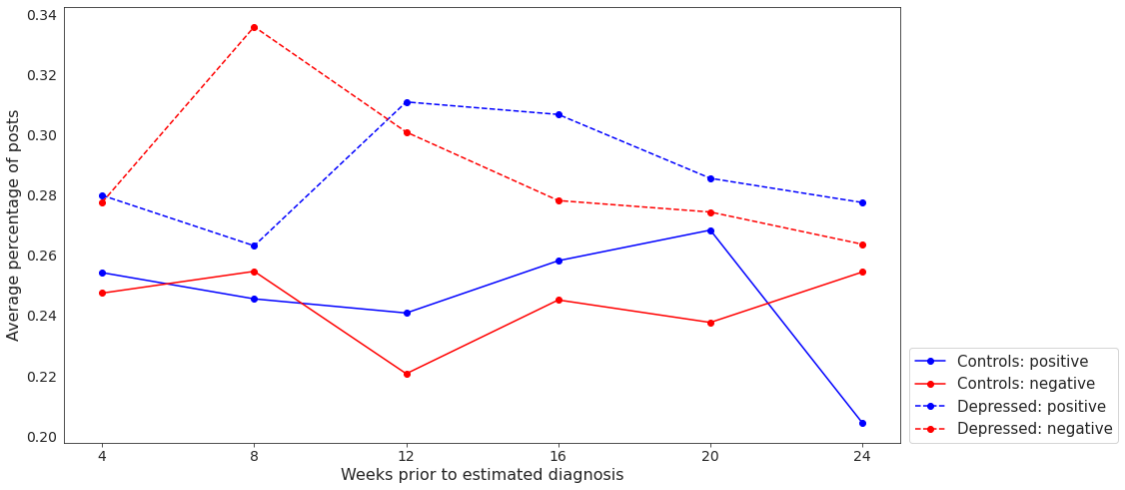
Change in the average percentage of positive and negative posts across 6 temporal bands: 0 to 4, 4 to 8, 8 to 12, 12 to 16, 16 to 20, and 20 to 24 weeks before the estimated diagnosis date (for a control user, this is the estimated diagnosis of its matched depressed user).

**Figure 4:**
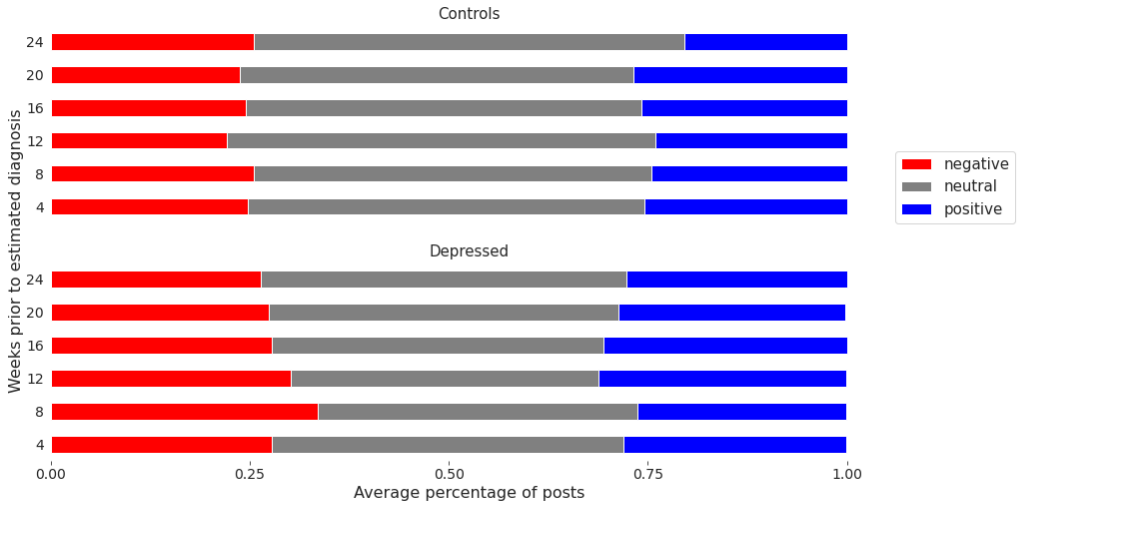
Average percentage of positive and negative posts per temporal band. Temporal bands include 0 to 4, 4 to 8, 8 to 12, 12 to 16, 16 to 20, and 20 to 24 weeks before the estimated diagnosis date (for a control user, this is the estimated diagnosis of its matched depressed user).

A sentiment analysis was then performed to complement the temporal experiment. We present the band-wise changes in sentiment for each class (Figures 3 and 4). It is observed that negatively charged posts for depressed users are less frequent as we approach the (estimated) diagnosis date, which may be deemed counterintuitive (Figure 3). However, it is also notable that depressed users’ posts were, on average, more negative than those of control users throughout the 24-week period (Figure 4). This aligns with previous studies that found a positive correlation between mental illness and negative sentiments [73].

We then sought to establish whether the diagnosis was associated with the sentiment of the post. The results of the logistic regression model (Table 15) indicate that there is a clear significant association between the diagnosis and the “sentimentality” of the post (*P*<.05), despite no apparent effect of temporality. Interestingly, the word count of a post appeared as a significant covariate of this model (*P*=.001), indicating that longer posts are slightly more likely to be classified as “sentimental,” irrespective of the depression status of the user.

Table 16 presents the results of the Multinomial Regression Model. Again, all effect size estimates were compatible with our inferences on the basis of a simpler logistic model. However, the multinomial analysis gives us an additional perspective: the effects of depression diagnosis are similar between positive and negative sentiments, with overlapping CIs statistically indistinguishable. This is the case despite the varying effects of other covariates, such as word count, which displays regression β coefficients of opposite signs in both sentiments (more words associate with negative posts, whereas fewer words associate with positive posts).

**Table 15:** Logistic regression results for predicting whether a post is neutral or not neutral.

Variable	β	Odds ratio	SE	*P* value
Depression diagnosis	0.163	1.177	0.035	<.001
Time to diagnosis	−0.004	0.996	0.013	.75
Post word count	0.040	1.041	0.012	.001
Interaction (diagnosis × time)	0.011	1.011	0.013	.41

**Table 16:** Multinomial regression results for predicting whether a post is positive or negative.

Sentiment and variable		β	Odds ratio	SE	*P* value
**Positive**					
	Depression diagnosis	0.190	1.209	0.047	<.001
	Time to diagnosis	0.015	1.015	0.016	.37
	Post word count	−0.070	0.932	0.019	<.001
	Interaction (diagnosis × time)	0.045	1.046	0.016	.006
**Negative**					
	Depression diagnosis	0.151	1.163	0.041	<.001
	Time to diagnosis	−0.019	0.981	0.016	.24
	Post word count	0.103	1.108	0.014	<.001
	Interaction (diagnosis × time)	−0.021	0.979	0.016	.18

## Discussion

### Principal Findings

We obtained evidence that LMs (particularly BERT-like models) can be used in preemptive mental health detection and analysis in longhand forums, even if they have room for improvement.

In our preemptive depression detection experiment, depressed and control subjects were placed in ratios of 1:1, 1:3, 1:5, and 1:10. The purpose was to simulate increasingly realistic settings in which most users were controls. In the balanced arrangement of 1:1, we obtained an *F*_1_-score of 0.738 using the MentalBERT LM. This is comparable with the works of Eichstaedt et al [14], de Choudhury et al [74], and Reece et al [19], who obtained *F*_1_-scores of 0.660, 0.680, and 0.650, respectively. This study provides evidence that LMs are more effective than existing methods for predicting depression in social media data before diagnosis.

Our temporal analysis suggested that the final 12 weeks (approximately 3 months) of posts before a depressed user’s estimated diagnosis date are likely to be the most indicative of their condition. Another broader interpretation is that LMs do not appear to improve with the addition of more data before 12-16 weeks. The BERT and MentalBERT obtained *F*_1_-scores of 0.726 and 0.715, respectively.

This is in contrast to a certain extent with the results of Eichstaedt et al [14], albeit using area under curve scores rather than *F*_1_-scores. Six months before the diagnosis date, 0.72 was obtained, and 3 months prior, 0.62 was obtained. From these results, it is difficult to draw clear conclusions because the results may be affected by the nature of the data and models used.

We also observed that posts made during the 4- to 8-week period before the user’s estimated diagnosis date are also pertinent. They exhibited more negative sentiment than posts made during any other 4-week period (up to 24 weeks before their estimated diagnosis date). This finding may be supportive of prior work that distinct changes in mood may be predictive of the onset of depression [75].

We were able to corroborate the importance of sentiment in the discourse of depressed users. We found that depressed users are approximately 1.18 times more likely to make a sentimental post than nondepressed users.

### Limitations

Constraints on our investigation primarily concern RSDD-Matched, where 70 depressed users make up a small sample. However, use 5-fold cross-validation to mitigate this and performed different experiments with various numbers of control users.

RSDD-Matched is derived from RSDD and RSDD-Time. As a result, the diagnosis dates of the users in RSDD-Matched are estimates only. Furthermore, posts made in mental health subreddits were deliberately elided from the RSDD and were not available for consideration by our machine classifiers.

### Conclusions

Using state-of-the-art LMs, this study posits how far the diagnosis of depression in a person with depressive traits can be determined in advance. With this knowledge, it may be possible to direct people with depression to physicians much sooner than they would otherwise. Moreover, perhaps more importantly, we have shown how these automatic NLP tools can serve to analyze the main traits arising from web-based posts.

We have also observed that the sentiment exhibited in web-based forum postings demonstrates good sensitivity in detecting depressive traits.

Further work may include a multimodal approach to the detection of people with depression in web-based forums such as Reddit. For example, along with the text of Reddit users’ posts, we might also consider the subreddits where they have upvoted and downvoted posts. The awards received or given may also indicate a user’s mental health. Such a study would, of course, be contingent on the ability to synthesize a suitable data set or source an existing one. Moreover, the use of temporal information such as temporal word embeddings [76] may enhance any multimodal approach.

Methods for gauging the severity of depression in web-based forum users should also be investigated. This might involve mining language features from user posts and observing how they correlate with ground-truth severity. Features of interest may include terms used in Linguistic Inquiry and Word Count dictionaries, sentiment, and emotion [77].

## Appendix

Multimedia Appendix 1 Reddit Self-reported Depression Diagnosis–Matched metadata and verbose results of the preemptive and temporal experiments.

## Abbreviations

ALBERT: A Lite Bidirectional Encoder Representations from Transformers
BERT: Bidirectional Encoder Representations from Transformers
LM: language model
MDD: major depressive disorder
NLP: natural language processing
RSDD: Reddit Self-reported Depression Diagnosis
SVM: support vector machine
TF-IDF: term frequency–inverse document frequency

## Data Availability

Information on the RSDD and RSDD-Time data sets used in this study, including their data access procedure, can be found on the web [78].
